# ORMDL3 regulates NLRP3 inflammasome activation by maintaining ER-mitochondria contacts in human macrophages and dictates ulcerative colitis patient outcome

**DOI:** 10.1016/j.jbc.2024.107120

**Published:** 2024-02-27

**Authors:** Jyotsna Sharma, Shaziya Khan, Nishakumari C. Singh, Shikha Sahu, Desh Raj, Shakti Prakash, Pamela Bandyopadhyay, Kabita Sarkar, Vivek Bhosale, Tulika Chandra, Jagavelu Kumaravelu, Manoj Kumar Barthwal, Shashi Kumar Gupta, Mrigank Srivastava, Rajdeep Guha, Veena Ammanathan, Uday C. Ghoshal, Kalyan Mitra, Amit Lahiri

**Affiliations:** 1 Pharmacology Division, CSIR-Central Drug Research Institute, Lucknow, India; 2 Academy of Scientific and Innovative Research (AcSIR), Ghaziabad, India; 3 Sophisticated Analytical Instrument Facility and Research Division, CSIR-Central Drug Research Institute, Lucknow, India; 4 Department of Gastroenterology, Sanjay Gandhi postgraduate institute of medical sciences, Lucknow, India; 5 Toxicology and Experimental Medicine Division, CSIR-Central Drug Research Institute, Lucknow, India; 6 Department of Transfusion Medicine, Kings George Medical University, Lucknow, India; 7 Molecular Parasitology and Immunology Division, CSIR-Central Drug Research Institute, Lucknow, India; 8 Lab Animal Facility, CSIR-Central Drug Research Institute, Lucknow, India

**Keywords:** ulcerative colitis, inlfammasome, mitochondrial-associated ER membrane, ORMDL3, mitochondria, interleukin-1β, cytokine

## Abstract

Genome-wide association studies in inflammatory bowel disease have identified risk loci in the orosomucoid-like protein 3/ORMDL sphingolipid biosynthesis regulator 3 (*ORMDL3*) gene to confer susceptibility to ulcerative colitis (UC), but the underlying functional relevance remains unexplored. Here, we found that a subpopulation of the UC patients who had higher disease activity shows enhanced expression of ORMDL3 compared to the patients with lower disease activity and the non-UC controls. We also found that the patients showing high *ORMDL3* mRNA expression have elevated interleukin-1β cytokine levels indicating positive correlation. Further, knockdown of ORMDL3 in the human monocyte-derived macrophages resulted in significantly reduced interleukin-1β release. Mechanistically, we report for the first time that ORMDL3 contributes to a mounting inflammatory response *via* modulating mitochondrial morphology and activation of the NLRP3 inflammasome. Specifically, we observed an increased fragmentation of mitochondria and enhanced contacts with the endoplasmic reticulum (ER) during ORMDL3 over-expression, enabling efficient NLRP3 inflammasome activation. We show that ORMDL3 that was previously known to be localized in the ER also becomes localized to mitochondria-associated membranes and mitochondria during inflammatory conditions. Additionally, ORMDL3 interacts with mitochondrial dynamic regulating protein Fis-1 present in the mitochondria-associated membrane. Accordingly, knockdown of ORMDL3 in a dextran sodium sulfate -induced colitis mouse model showed reduced colitis severity. Taken together, we have uncovered a functional role for ORMDL3 in mounting inflammation during UC pathogenesis by modulating ER-mitochondrial contact and dynamics.

Inflammatory bowel disease (IBD) that encompasses of ulcerative colitis (UC) and Crohn’s disease is a chronic inflammatory condition of the gastrointestinal tract. It is characterized by excessive inflammatory response that leads to the disruption of gut tissues and altered intestinal permeability (1). Multiple genome-wide association studies (GWAS) have been performed to understand IBD susceptibility and more than 200 risk loci including in orosomucoid-like protein 3/ORMDL sphingolipid biosynthesis regulator 3 (*ORMDL3*) loci have been identified (2, 3). In our efforts to define the function of GWAS associated unexplored genes in the context of UC pathogenesis, we focussed on ORMDL3 in the present study.

GWAS studies have strongly linked multiple genetic variants on the regulatory locus of *ORMDL3* harboring 17q21 to be associated with diseases such as IBD, diabetes, and asthma (4, 5). These polymorphisms were shown to perturb *ORMDL3* gene expression, and risk variants associated with asthma had increased *ORMDL3* expression contributing to risk of triggering childhood asthma (6, 7). Functional analysis revealed that overexpression of ORMDL3 in lung epithelial cells leads to activation of unfolded protein response in the endoplasmic reticulum (ER) and metalloproteases such as matrix metalloproteinase 9, ADAM8 involved in the airway remodeling (8, 9, 10). In case of UC, multiple independent studies conducted in separate cohorts have identified SNPs on the regulatory locus of ORMDL3 (2, 3, 11, 12) without any functional validation.

ORMDL gene family members (ORMDL1, ORMDL2, and ORMDL3) encode for transmembrane proteins present on ER (13, 14). ORMDL homologous present on chromosomes 2, 12, and 17 share amino acid identities of about 80% (14). Further, ORMDL proteins are involved in the regulation of *de novo* sphingolipid synthesis by inhibiting serine-palmitoyl transferase, the first-rate limiting enzyme in the sphingolipid synthesis pathway (15). Deletion of *ORMDL* genes, especially *ORMDL3* in mice shows elevated levels of sphingolipids and ceramide (16, 17, 18). Other physiological functions related to ORMDL3 include regulating ER-unfolded protein response, cell death, calcium response, and cytokine/chemokine secretion. However, the relevance of ORMDL3 in UC pathogenesis remains underexplored till date.

We have recently showed that mitochondrial dysfunction decides the pathogenesis of UC as mitochondrial quality control pathways regulate immune response in the intestinal mucosa (19)]. Mitochondria additionally forms close contact with other cellular organelles including ER (20)] The proteins residing at ER and mitochondria make dynamic interactions forming multiorganelle domains referred to as mitochondrial-associated ER membranes (MAMs) (21). Multiple inflammation-associated proteins like MAVS, NLRP3 gets localized to MAMs. NLRP3 migrates from the ER to MAMs and mitochondria during inflammasome activation and subsequently leads to mature IL-1β and IL-18 production (22).

We observed that a subpopulation of the UC patients with more disease score in our cohort had enhanced expression of *ORMDL3*, and interleukin-1β (IL-1β) secretion. We hypothesized that mucosal ORMDL3 level could dictate IL-1β secretion in the UC patients. We report here for the first time that ORMDL3 accumulates in the MAM and mitochondria leading to increased ER-mitochondria tethering during inflammatory conditions. This tethering subsequently allows heightened NLRP3 inflammasome docking to MAM producing more matured IL-1β. By mass spectrometry analysis we found Fis1 (MAM associated proteins) interacts with ORMDL3, which might regulate mitochondrial dynamics leading to increased mitochondrial fission (23). We have unveiled that *ORMDL3* overexpression leads to increased ER-mitochondria contact formation and mitochondrial fission. Further, we found that the reduction of ORMDL3 expression level in the mice model of colitis reduces inflammation as well as disease severity.

## Results

### ORMDL3 expression in colonic biopsy specimens in the UC patient is strongly correlated with IL-1β secretion and disease score

**Figure 1:**
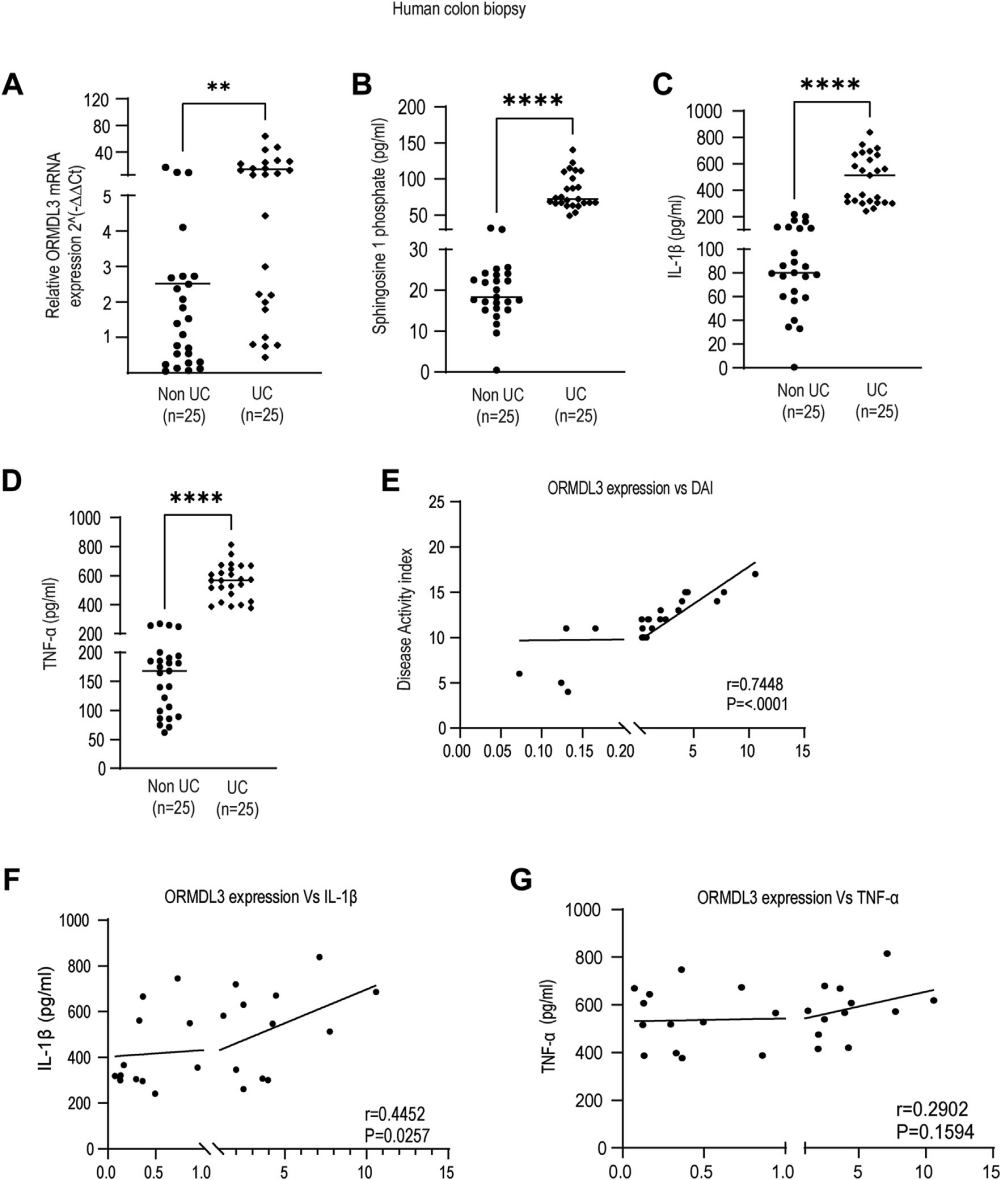
***ORMDL3* level in the ulcerative colitis patients shows positive correlation with IL-1β but not with TNF-**α **and sphingosine -1-phosphate.** Total RNA was isolated from ulcerative colitis (UC) and non-UC control patient’s colon biopsy samples to check (*A*) relative mRNA expression of ORMDL3 in UC (n = 25) and non-UC (n = 25) colon using gene specific primer, Beta2 macroglobulin was used as control. UC and non-UC intestinal samples were lyzed and supernatants were subjected to ELISA analysis to assess (*B*) level of sphingosine-1-P in UC and non-UC control (n = 25), (*C*) IL-1β level (n = 25). *D*, TNF-α level (n = 25). *E*, correlation analysis between *ORMDL3* mRNA fold expression (2^∧^- Δ Ct) and disease activity index of respective UC patients showing positive significant correlation (n = 25). *F*, correlation analysis between *ORMDL3* mRNA fold expression (2^∧^- Δ Ct) and IL-1β of the respective patients showing positive significant correlation (n = 25). *G*, graph showing correlation between ORMDL3 mRNA fold expression (2^∧^- Δ Ct) and TNF-α level in UC patient colon showing nonsignificant correlation (n = 25). In the correlation graph, “r” denotes correlation coefficient and “*p*” value less than 0.05 indicates significant correlation. Mean ± SEM; ∗∗*p* < 0.01; ∗∗∗∗*p* < 0.0001; as determined by 2-tailed *t* test. IL, interleukin; ORMDL3, orosomucoid-like protein 3; TNF, tumor necrosis factor; UC, ulcerative colitis.

**Supporting Figure S1:**
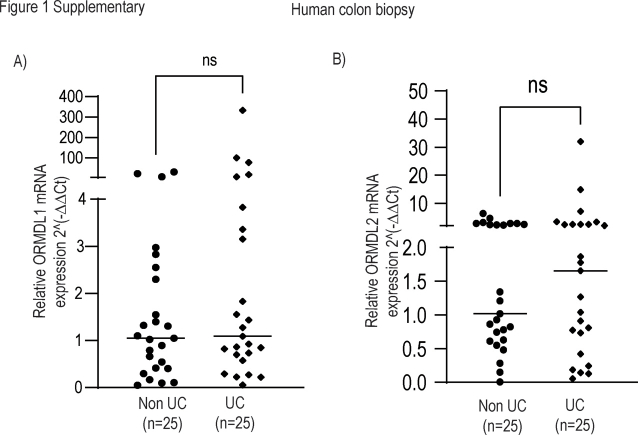
**ORMDL1 and ORMDL2 expression is unchanged in the ulcerative colitis patients.***A*, relative mRNA expression of ORMDL1 in UC (n = 25) and non-UC (n = 25) colon. *B*, relative mRNA expression of ORMDL2 in UC (n = 25) and non-UC colon (n = 25) using gene specific primer, Beta2 macroglobulin was used as control. Mean ± SEM, significance was determined by 2-tailed t test. “ns” denotes non-significant.

SNPs in the *ORMDL3* locus have been found to be associated with UC pathogenesis (24). Also, ORMDL3 is known as a negative regulator of sphingolipid synthesis (25). Hence to understand the role of ORMDL3 in UC pathogenesis, we initiated our study by measuring the expression levels of *ORMDL3* in the colon biopsy specimens of the UC patients and non-UC controls. *ORMDL3* expression was first checked at the mRNA level and we found that *ORMDL3* was significantly upregulated in the colon biopsy samples of the UC patients in comparison to the non-UC controls (Fig. 1*A*). As ORMDL3 is known to negatively regulate sphingolipid biosynthesis pathway in asthma, we checked sphingosine 1 phosphate (S1P) in the intestine of UC patients. We found that the UC patients have high S1P level as shown in the previous studies (26) and thereby making us conclude that ORMDL3 does not regulate S1P production in the intestine (Fig. 1*B*). Proinflammatory cytokine secretion is enhanced in the UC patients and contribute to chronic inflammation in the intestine. Accordingly, level of IL-1β and tumor necrosis factor (TNF)-α in the UC intestine samples were found to be significantly higher compared to the non-UC controls (Fig. 1, *C* and *D*). We have collected disease activity index (DAI) scores (19) of all the patients whose samples were used for our study. DAI score denotes disease status of the patients. We observed that the patients having high DAI score had high level of ORMDL3 expression showing positive correlation with the disease (Fig. 1*E*). The expression level of *ORMDL3* in the intestinal sample of UC patients also shows positive correlation of IL-1β with *ORMDL3* expression (Fig. 1*F*) while TNF-α does not show significant correlation with *ORMDL3* expression in the patients (Fig. 1*G*). Hence, our results showed that ORMDL3 plays important role in UC pathogenesis and might regulate IL-1β secretion in the gut mucosa. ORMDL family members ORMDL1 and ORMDL2 present on ER shares 80% identity with ORMDL3, but they are not associated with UC. We next measured their transcript levels in the intestinal biopsy samples. We observed no significant difference in the relative mRNA levels of *ORMDL1* and *ORMDL2* in the intestinal samples of UC patients compared to the non-UC controls. (Fig. S1, *A* and *B*).

### ORMDL3 acts downstream of LPS and regulates IL-1β secretion from human macrophages

**Figure 2:**
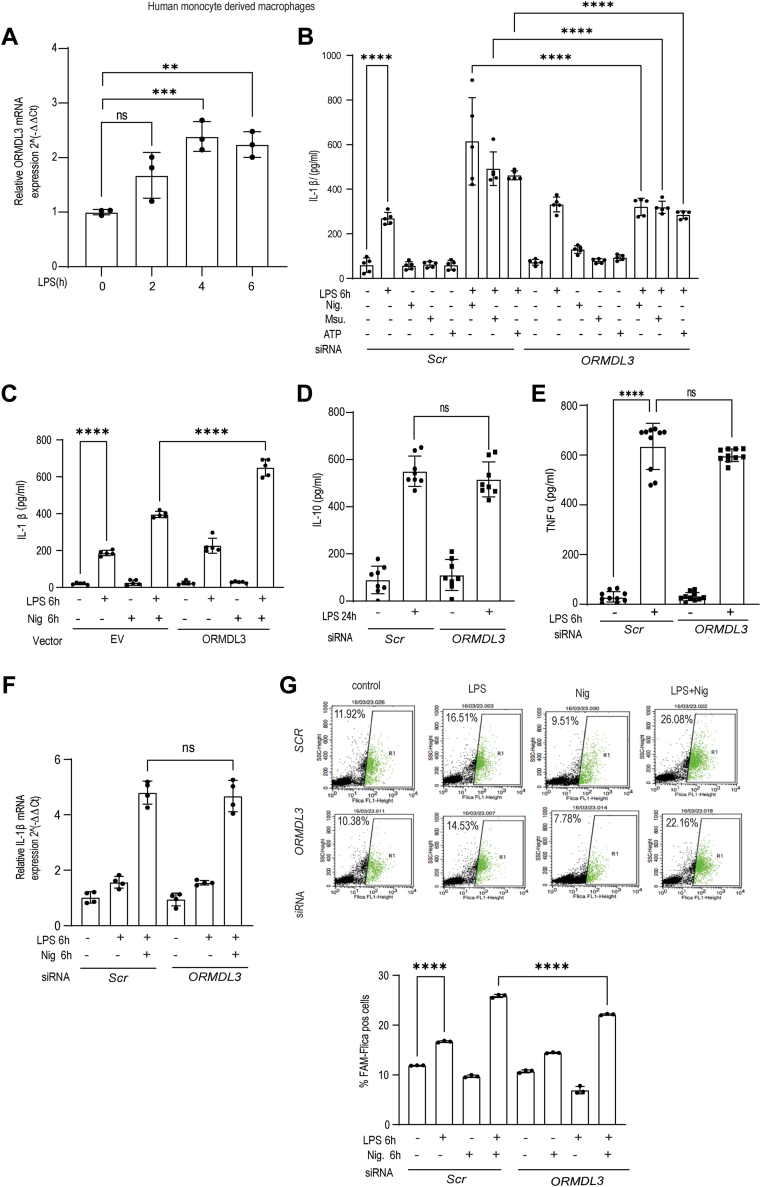
**Enhanced ORMDL3 expression level leads to more IL-1β production.***A*, human MDMs were treated with LPS (200 ng/ml) for indicated times and assessed for mRNA expression of *ORMDL3* at different time points (n = 3 donors, similar result obtained for an additional n = 3 donor). *B*, human MDM cells were transfected with scrambled or siORMDL3 for 24 h, then stimulated with LPS, ATP, MSU, and nigericin alone and LPS along with MSU (150 μg/ml) and nigericin (10 μM) for 6 h and ATP (5 mM) for 30 min after 5 h of LPS treatment and analyzed for IL-1β secretion in the cell supernatants (n = 5 donor). *C*, human MDM cells were transfected with *ORMDL3* expressing full-length plasmid and empty vector for 24 h, then stimulated with LPS(200 ng/ml) and nigericin for 6 h and analyzed for IL-1β secretion in cell supernatant (n = 6, similar result obtained for additional n = 6 donor). Human MDM were transfected with scrambled or siORMDL3 for 24 h, treated with LPS (200 ng/ml) for another 24 h and analyzed for (*D*) IL-10 secretion in cell supernatant (n = 8 donor, similar result was obtained for additional n = 8 donor) and (*E*) TNF-α secretion in the cell supernatant (n = 10 donor, similar result was obtained for additional n = 10 donor). Human MDM cells were transfected with scrambled and siORMDL3 for 24 h, treated with LPS (200 ng/ml) and nigericin for 6 h and analyzed for (*F*) relative IL-1β mRNA fold by qRT-PCR (n = 4 donor, similar result obtained for additional n = 4 donor) and (*G*) Caspase-1 activation by flow cytometry (n = 3 donor, similar result for additional n = 3 donor). Mean ± SEM; ∗*p* < 0.05, ∗∗*p* < 0.01; ∗∗∗*p* < 0.001; ∗∗∗∗*p* < 0.0001 as determined by one-way ANOVA. EV, empty vector, ns denotes non-significant. IL, interleukin; LPS, lipopolysaccharide; MDM, monocyte-derived macrophage; MSU, monosodium urate; ORMDL3, orosomucoid-like protein 3; qRT-PCR, quantitative reverse transcription PCR; TNF, tumor necrosis factor.

**Supporting Figure S2:**
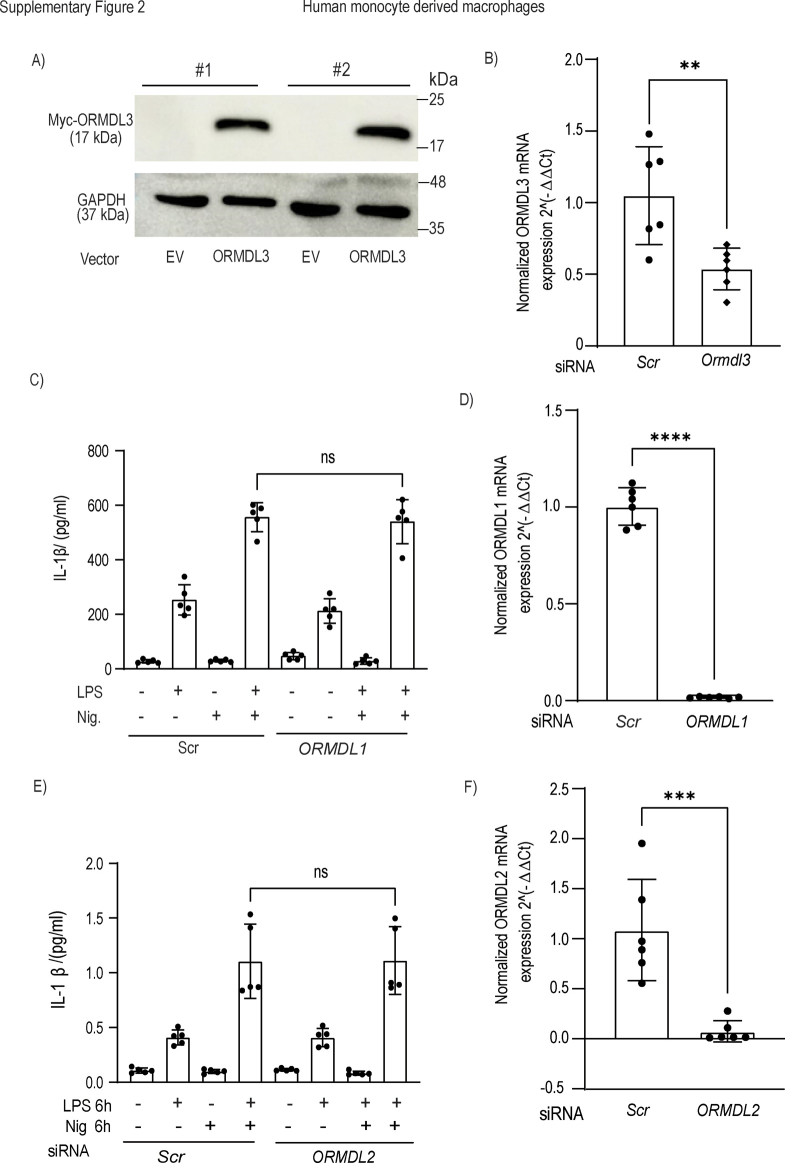
**ORMDL1 and ORMDL2 expression does not affect IL-1β level in human MDMs.***A*, human MDM cells were transfected with full length Myc tagged ORMDL3 for 24 h and assessed for overexpression efficiency of myc-ORMDL3 in human monocyte derived macrophages by western blot. GAPDH was used as loading control. *B*, human MDMs were transfected with scrambled or siORMDL3 for 24 h and assessed for relative mRNA fold change of ORMDL3 showing knockdown efficiency by qRT PCR (n = 6 donor). *C*, human MDM were transfected with scrambled or siORMDL1 for 24 h, then stimulated with LPS ( 200 ng/ml) and Nigericin for 6 h and analysed for IL-1β secretion in the cell supernatant of (n = 5 donor). *D*, human MDMs were transfected with scrambled or siORMDL1 for 24 h and assessed for relative mRNA fold change of ORMDL1 showing knockdown efficiency by qRT PCR (n = 6 donor). *E*, human MDM were transfected with scrambled or siORMDL2 for 24 h ,then stimulated with LPS (200 ng/ml) and Nigericin for 6 h and analysed for IL-1β secretion in the cell supernatant of n = 5 donor. *F*, human MDMs were transfected with scrambled or siORMDL2 for 24 h and assessed for relative mRNA fold change of ORMDL2 showing knockdown efficiency by qRT PCR (n = 6 donor). Mean ± SEM, ∗∗*P* < 0.01, ∗∗∗*P* < 0.001, ∗∗∗∗*P* < 0.0001 as determined by 1-way ANOVA for Figure 2, C and *E* and 2-tailed t test for rest of the figure. “ns” denotes non-significant.

Migrated blood derived monocytes and intestinal macrophages in the UC patient’s intestine produce IL-1β (26). We thereby went on to relate ORMDL3 expression with inflammatory outcomes in the primary human blood monocyte-derived macrophages (MDMs). To activate NLRP3 inflammasome, cells should be treated with primary signal (lipopolysaccharide [LPS] is used) and secondary signals including ATP, monosodium urate (MSU), and nigericin. ATP, MSU, and nigericin are important for the assembly and activation of the NLRP3 inflammasome. In these primary cells, bacterial ligand LPS stimulation led to *ORMDL3* upregulation at mRNA level in a time-dependent manner (Fig. 2*A*). Additionally, significant decrease in the IL-1β level was noted after *ORMDL3* knockdown in the human MDMs stimulated with LPS along with nigericin, MSU, and ATP (Fig. 2*B*). *ORMDL3* knockdown efficiency is shown in Fig. S2*B*. Furthermore, *ORMDL3* overexpression showed high IL-1β secretion from the human MDM cells (Fig. 2*C*) validating our patient data. ORMDL3 overexpression efficiency is shown in Fig. S2*A*. In contrast, *ORMDL3* knockdown had no effect on IL-10 and TNF-α production (Fig. 2, *D* and *E*). Mechanism of IL-1β release has three steps, at first IL-1β is produced as pro IL-1β, in next step biologically active IL-1β is produced by caspase 1 and finally it is released in extracellular milieu in the third step. Hence, we asked at what step does ORMDL3 regulates IL-1β release. LPS treatment increased IL-1β expression; however, mature IL-1β is secreted after NLRP3 inflammasome mediated caspase1 activation. To understand the effect of ORMDL3 on IL-1β production, we next examined *IL-1β* mRNA level in human MDM cells after *ORMDL3* knockdown with LPS and nigericin stimulation and observed no difference. Since ORMDL3 did not affect *IL-1β* mRNA level (Fig. 2*F*), we next measured caspase1 activation in the human MDM cells. *ORMDL3* depleted cells when treated with LPS and nigericin had significantly reduced caspase1 activity compared to the control cells (Fig. 2*G*). We found that *ORMDL3* depletion does not show enhanced cell death in the LPS stimulated MDMs. *ORMDL1* and *ORMDL2* knockdown had no effect on IL-1β expression (Fig. S2, *C* and *E*). ORMDL1 and ORMDL2 knockdown efficiency is shown in the Fig. S2, *D* and *F*.

### ORMDL3 is localized to MAM and mitochondrial fractions to regulate ER-mitochondria contacts as well as NLRP3 inflammasome activation

**Figure 3:**
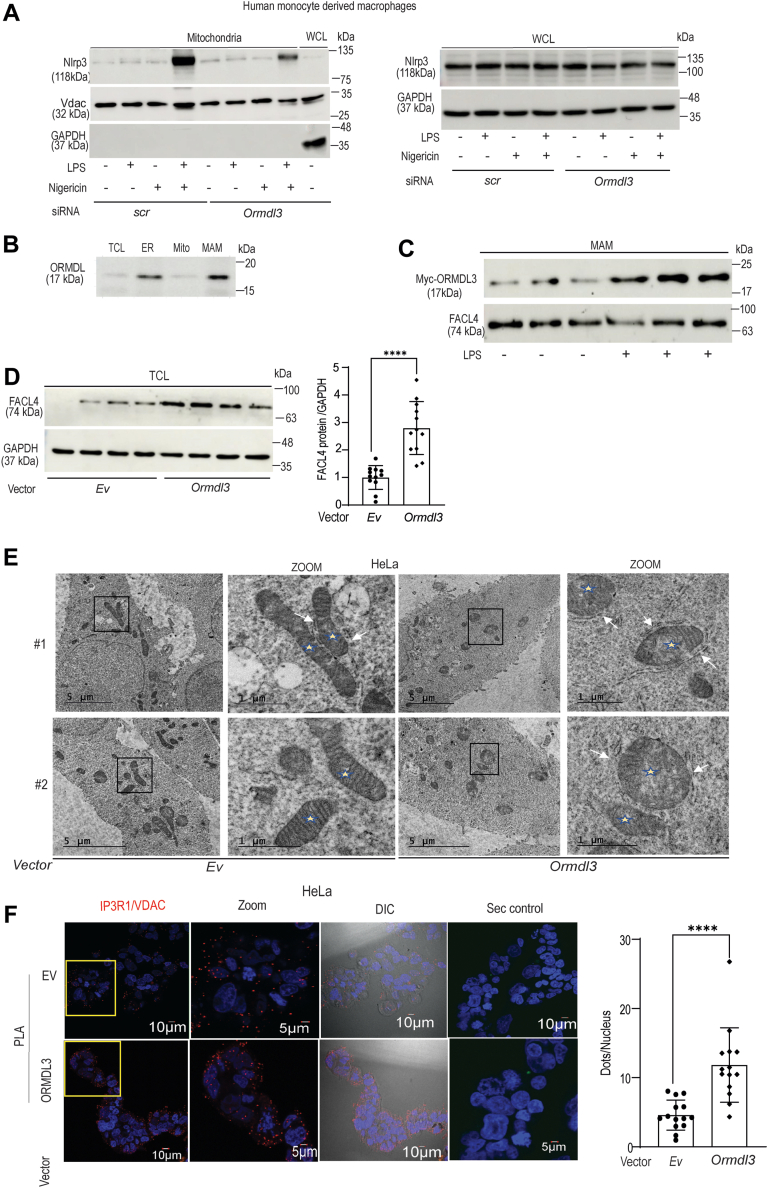
**ORMDL3 upregulation enhances ER-mito contact formation and regulates NLRP3 inflammasome activation.***A*, MDM cells were transfected with scrambled or siORMDL3 for 24 h and were treated with LPS (200 ng/ml) with nigericin for 6 h (10 μM). Mitochondria was isolated and NLRP3 expression was assessed by Western blotting in mitochondrial fractions and whole cell lysates (WCL) separately. Shown are representative blot from one donor out of total four donors tested. GAPDH and VDAC1 were used as loading control for whole cell lysates and mitochondrial fractions, respectively, (*B*) subcellular fractions (ER, mitochondria, and MAM) from HeLa cells were isolated by ultracentrifugation and assessed for ORMDL expression in different fractions by Western blotting (shown is representative blot with additional five repeats). *C*, HeLa cells were transfected with myc-tagged ORMDL3 plasmid for 24 h, treated with LPS (250 ng/ml) for 6 h then subcellular fractions were isolated and assessed for myc-ORMDL3 expression in MAM subcellular fraction using myc antibody and MAM marker FACL4 used as control, similar results were obtained for additional n = 3 donors. *D*, HeLa cells were transfected with myc-tagged ORMDL3 plasmid or empty vector (Ev) for 24 h and MAM marker FACL4 expression in total cell lysate (TCL) is shown; GAPDH was used as loading control. A summary graph of a densitometry analysis after normalization with GAPDH (from three blots). *E*, HeLa cells were transfected with *myc-ORMDL3* full-length plasmid or empty vector (Ev) and assessed for ER-mito contacts by transmission electron microscopy (TEM). Representative TEM images are showing ER-mitochondria contacts, the scale bar represents 5 μm and 1 μm.(*White arrow* shows ER-mito contact, a total of 20 cells were imaged for both transfected and control). *F*, HeLa cells were transfected with *myc-ORMDL3* full-length plasmid or empty vector (Ev) for 24 h and assessed for ER-mitochondria contacts by *in situ* proximity ligation assay (PLA). Representative confocal images are shown with zoomed images and DIC images and a negative control with secondary antibody only (Sec control). PLA dots shows IP3R1-VDAC1 complex as *red dots* at ER-mito contact and PLA dots per nucleus quantitated (a total of 500–600 cells were acquired in two independent experiments). The scale bar represents 10 μm and 5 μm. ∗∗∗∗*p* < 0.0001 as determined by 2-tailed *t* test. DIC, differential interference contrast; ER, endoplasmic reticulum; FACL4, fatty-acid CoA ligase 4; LPS, lipopolysaccharide; MAM, mitochondrial-associated ER membrane; MDM, monocyte-derived macrophages; ORMDL3, orosomucoid-like protein 3; VDAC, voltage-dependent anion channel.

**Supporting Figure S3:**
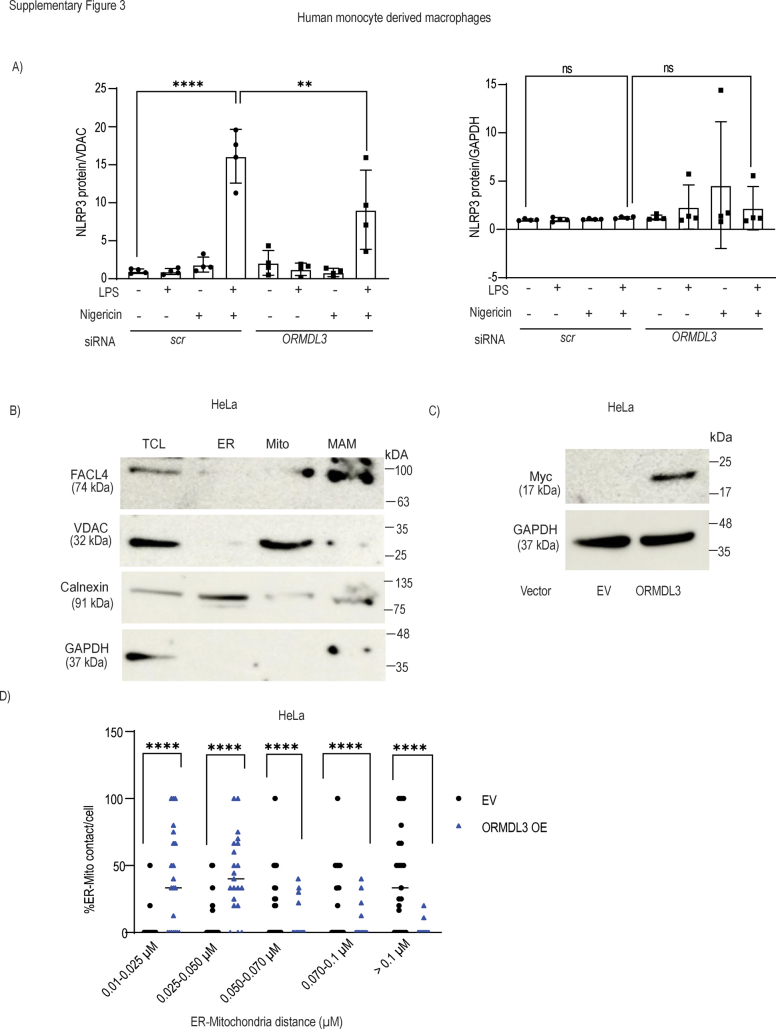
**ORMDL3 overexpression decreases ER-mito distance and promotes NLRP3 localization to mitochondria not total NLRP3 expression.***A*, human MDMs were transfected with scrambled or siORMDL3 for 24 h, treated with LPS (200 ng/ml) along with MSU nigericin (10 μM) for 6 h. Summary graph showing western blot densitometry analysis of NLRP3 localization to mitochondria and total NLRP3 level in total cell lysates from 4 donors. *B*, subcellular fractions including ER, Mitochondria, and MAM were isolated from HeLa cells and assessed for purity of ER, mitochondria and MAM fractions by Western blotting using antibodies against FACL4 (MAM marker) or calnexin (ER marker), or VDAC (mitochondrial marker). *C*, HeLa cells were transfected with myc tagged *ORMDL3* full length plasmid or Empty vector (Ev) for 24 h and assessed for ORMDL3 overexpression efficiency in total cell lysate by western blotting using myc antibody, GAPDH was used as loading control. *D*, ER-mito contact distance quantified after acquiring images by transmission electron microscopy(TEM) (A total of 20 cells were quantified for ER-mito contact distance in Ev and myc-ORMDL3 transfected cells). Mean ± SEM; ∗∗*P* < 0.01; ∗∗∗∗*P* < 0.0001 as determined by one-way ANOVA for Figure 2*A* and two-way ANOVA for Figure 2D. ns denotes non-significant.

As our previous results showed that *ORMDL3* depletion reduces caspase-1 activation but does not affect *IL-1β* mRNA level, we next focused on the assembly and activation of NLRP3 inflammasome after *ORMDL3* knockdown. NLRP3 is mostly present in the cytosol and ER in inactive state, upon stimulation it gets recruited to the mitochondria and mitochondria associated membrane (27). *ORMDL3* depleted human MDMs when stimulated with LPS in the presence of Nigericin showed reduced localization of NLRP3 to the mitochondria (Figs. 3*A* and S3*A*). However total NLRP3 level remained unchanged (Figs. 3*A* and S3*A*) suggesting that ORMDL3 regulate NLRP3 localization to mitochondria, but does not affect total NLRP3 level.

Due to limitation of primary human blood-derived MDM, we used a cell line for the subcellular fractionation experiments. We observed that in the intestine, ORMDL3 had no effect on the sphingolipid production. To better understand the sphingolipid independent role of ORMDL3, HeLa cells were used for the subcellular fractionations and few overexpression studies, as previous studies with ORMDL3 knockdown in the HeLa cell showed no effect on the ceramide production. Commercial antibody against ORDML3 is not available. We analyzed the localization of endogenous ORMDL family proteins (ORMDL1/2/3) during basal condition in different subcellular fractions isolated from the HeLa cells using antibody which recognizes ORMDL1/2/3 (termed as ORMDL). Interestingly, we found that ORMDL was present in the MAM and mitochondria in addition to its established ER localization (Fig. 3*B*). Purity of the subcellular fractions was confirmed by specific organelle markers like fatty-acid CoA ligase 4 (FACL4) (for MAM), voltage-dependent anion channel (VDAC) (for MAM and mitochondria), and calnexin (for ER) (28) as shown in the Fig. S3*B*. ER-mitochondria contact sites serve as an important signaling platform which harbors several proteins regulating mitochondrial dynamics and immune response including MFN2, Drp1, NLRP3, and so on. Hence, we wanted to check the expression of ORMDL3 in mitochondria associated membrane to understand its role over mitochondrial function and immune response. ORMDL3 localization was found to be increased in the MAM after *myc*-*ORMDL3* overexpressing HeLa cells were stimulated with LPS (Fig. 3*C*). We hypothesized that aberrant NLRP3 inflammasome activation after ORMDL3 overexpression might be due to altered ER-mitochondria contact formation. The localization of ORMDL3 in the MAM fraction suggested that ORMDL3 might regulate MAM integrity or ER–mitochondria distance (29). Hence, we checked the extent of MAM formation after *ORMDL3* overexpression. The level of MAM marker protein long-chain-FACL4 increased in the total cell lysate with ORMDL3 overexpression indicating increased MAM formation (Fig. 3*D*). *ORMDL3* overexpression efficiency is shown in Fig. S3*C*. Further on, to confirm our result we analyzed the effect of *ORMDL3* overexpression on ER–mitochondria contact formation by transmission electron microscopy (TEM) and proximity ligation assay. TEM results clearly showed that the *ORMDL3* overexpressing cells had increased number of closely apposed ER and mitochondria contacts at a distance of less than 40 nm while in the control cells number of ER–mitochondria contacts were significantly low (Figs. 3*E* and S3*D*). The extent of ER–mitochondria contact formation additionally can be measured by interaction of IP3R1-GRP75-VDAC (30). Proximity ligation assay (PLA) was performed next to validate the TEM results to check ER–mitochondria interaction using antibodies against ER resident protein IP3R1 and mitochondria–specific protein VDAC (PLA showed red puncta only after colocalization; single stains will not show any color). The results clearly demonstrated increased ER-mitochondria contact formation after ORMDL3 overexpression (Fig. 3*F*). Hence, our result revealed that ORMDL3 localizes in the MAM and enhances ER–mitochondria contact formation which might enhance NLRP3 inflammasome activation and IL-1β production.

### ORMDL3 overexpression alters mitochondrial dynamics and leads to mitochondrial dysfunction

**Figure 4:**
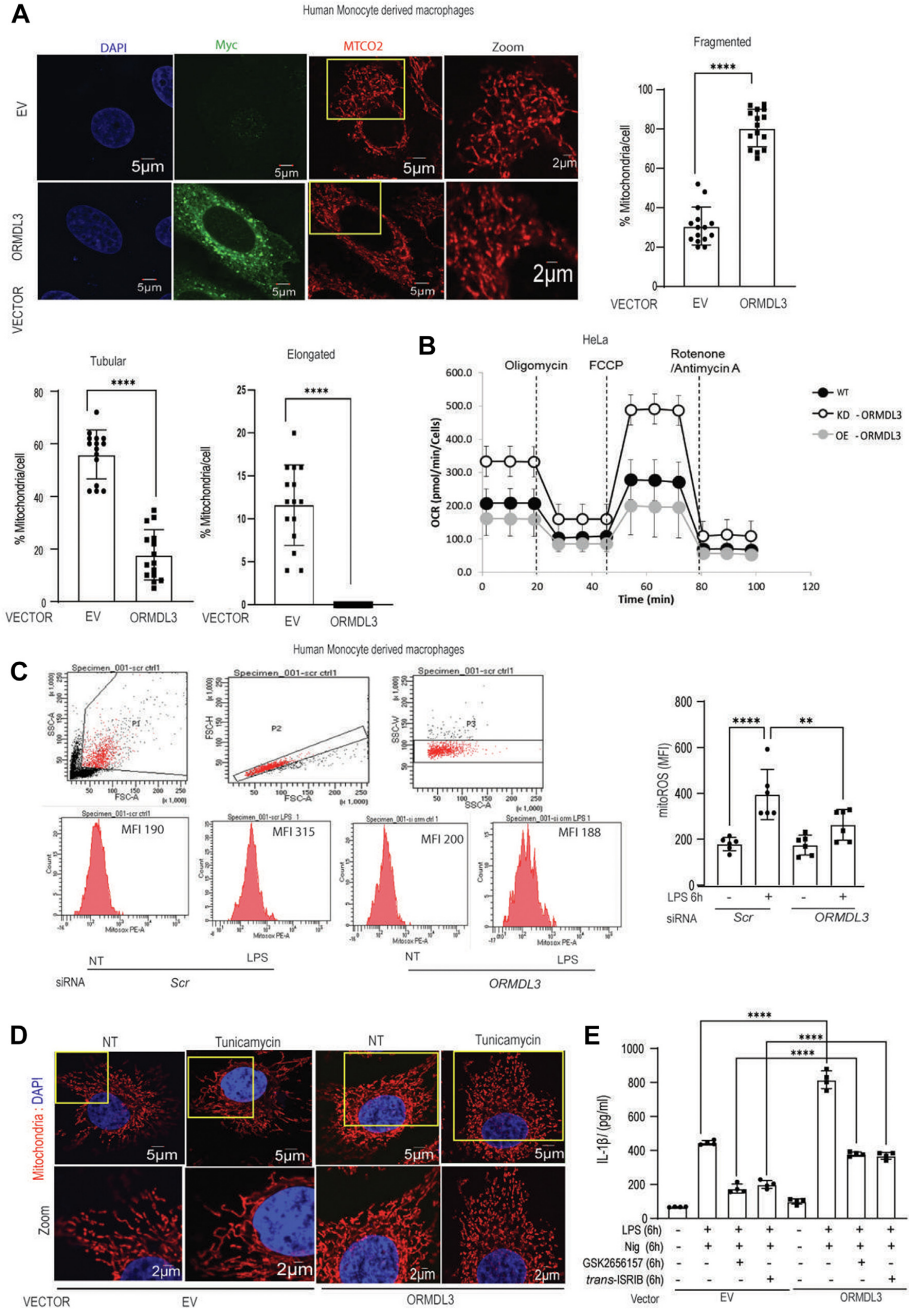
**ORMDL3 overexpression alters mitochondrial morphology and causes mitochondrial dysfunction.***A*, human MDMs were transfected with myc-tagged *ORMDL3* and empty vector for 24 h and stained with MTCO2(*red*) as mitochondrial marker, myc (*green*) as ORMDL3 marker, and DAPI (*blue*) to stain nucleus, representative confocal images shows mitochondrial morphology. Total 50 to 60 cells were acquired in three independent experiments and summary graph showing mitochondrial morphology quantified for control and ORMDL3 overexpression to analyze mitochondrial morphology. The scale bar represents 5 μm and 2 μm. *B*, HeLa cells were transfected with scrambled, and siORMDL3, as well as *ORMDL3* full-length expressing plasmid for 24 h and assessed for oxygen consumption rate (OCR), measured following mitochondrial stress after addition of oligomycin A, FCCP, and Rotenone/Antimycin A in control, ORMDL3 overexpression and ORMDL3 knockdown. *C*, human MDMs were transfected with scrambled or siORMDL3 for 24 h followed by LPS (200 ng/ml) treatment for 6 h and Mito ROS production was detected by flow cytometry. Histograms representing Mitosox fluorescence in different groups and bar graph representing mitochondrial ROS production. (n = 6 donor, similar result obtained for additional n = 3 donor). *D*, human MDM cells were transfected with empty vector or full-length *ORMDL3* expressing plasmid, treated with ER stress inducer tunicamycin for 6 h and stained with MTCO2(*red*) as mitochondrial marker, and DAPI (*blue*) to stain nucleus, representative confocal images show mitochondrial morphology. (a total of 20–30 cells were acquired in two independent experiments). The scale bar represents 5 μm and 2 μm. *E*, human MDM cells were transfected with ORMDL3 for 24 h and treated with LPS (200 ng/ml) along with nigericin with and without ER stress inhibitors *trans*-ISRIB (200 ng/ml) and GSK2656157 (10 μM) for 6 h and analyzed for IL-1β secretion by ELISA (n = 4 donor, similar result obtained for additional n = 4 donor). Mean ± SEM; ∗∗*p* < 0.01; ∗ ∗∗∗∗*p* < 0.0001 as determined by 2-tailed *t* test for A and one-way ANOVA for rest of the Figures. DAPI, 4′,6-diamidino-2-phenylindole; ER, endoplasmic reticulum; FCCP, carbonyl cyanide-p trifluoromethoxyphenylhydrazone; IL, interleukin; ISRIB, integrated stress response inhibitor; LPS, lipopolysaccharide; MDM, monocyte-derived macrophage; ORMDL3, orosomucoid-like protein 3.

**Supporting Figure S4:**
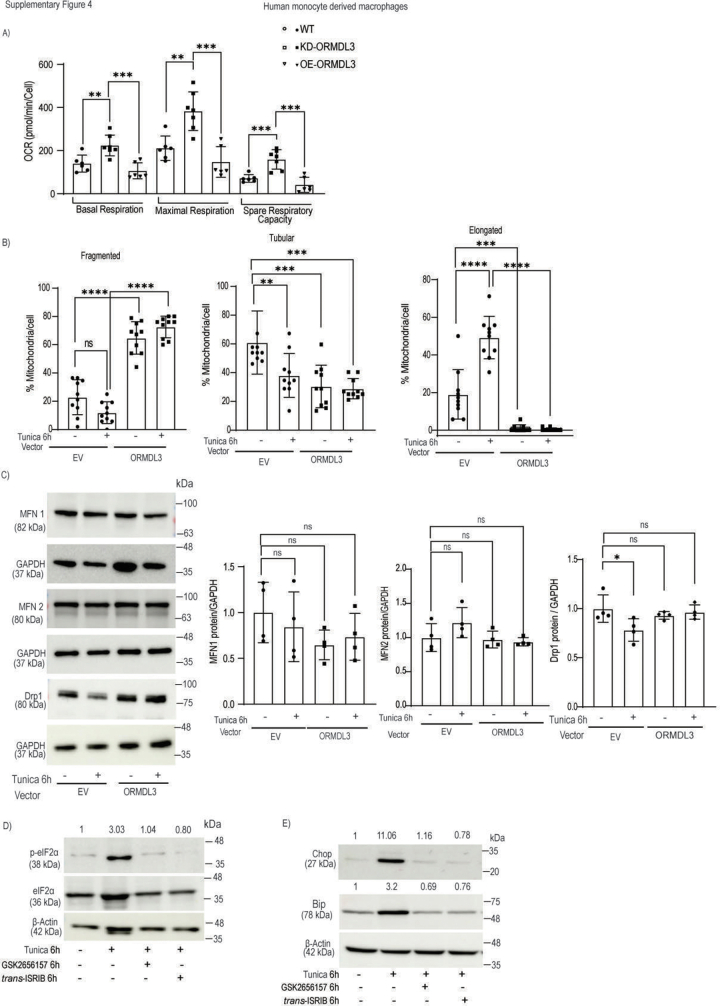
**ORMDL3 regulates mitochondrial dynamics independent of ER stress.***A*, HeLa cells were transfected with scrambled or siORMDL3 or ORMDL3 full length expressing plasmid for 24 h. Summary graph showing maximal, basal respiration and spare respiratory capacity following mitochondrial stress after addition of oligomycin A, FCCP and rotenone/ontimycin A in control, ORMDL3 overexpression and ORMDL3 knockdown cells. *B*, human MDM cells were transfected with empty vector (EV) or full length *ORMDL3* expressing plasmid, treated with ER stress inducer tunicamycin for 6 h and stained with MTCO2 (*red*) as mitochondrial marker, and DAPI (*blue*) to stain nucleus. Summary graph is plotted showing mitochondrial morphology. *C*, human MDM cells were transfected with empty vector (EV) or full length *ORMDL3* expressing plasmid and treated with ER stress inducer tunicamycin for 6 h. Cells were lysed and mitochondrial du = ynamics associated protein MFN-1, MFN-2 and Drp1 proteins expression was checked and representtaive western blot is shown. GAPDH was used as loading control. Summary graph test protein expression normalized to control protein is shown on *right*. Human MDMs were treated with tumicamycin with or without inhibitors *trans*-ISRIB and GSK2656157 and cells were lysed and ER stress marker proteins expresseion was checked for *D*, ER stress maker proteins phospho eIF2 alpha and total eiF2 alpha, β actin and *E*) Chop and Bip by western blot. β actin was used as loading control. Values above band denote nomralzed expression level of test protein compared to the control protein. Mean ± SEM; ∗*P* < 0.05, ∗∗*P* < 0.01; ∗∗∗*P* < 0.001, ∗∗∗∗*P* < 0.0001 as determined by one-way ANOVA. ns denotes non-significant.

Our results till now showed that *ORMDL3* overexpression leads to increased contact formation between ER and mitochondria. ER–mitochondria contact regulates mitochondrial function, dynamics (31) and NLRP3 inflammasome assembly and activation (28) making us explore the effect of ORMDL3 on mitochondrial dynamics. Human MDMs with *ORMDL3* overexpression exhibited increased mitochondrial fission (Fig. 4*A*). To understand the role of ORMDL3 in mitochondrial dysfunction we next checked oxygen consumption rate (OCR) in the HeLa cells with *ORMDL3* overexpression and knockdown. OCR decreased in cells with *ORMDL3* overexpression while *ORMDL3*-depleted cells have high OCR which indicates that upregulation of this gene leads to mitochondrial dysfunction (Fig. 4*B*). Basal respiratory capacity, maximal respiratory capacity, as well as spare respiratory capacity was also significantly reduced in the cells with *ORMDL3* overexpression when compared to cells depleted of ORMDL3 as well as control cells (Fig. S4*A*). Collectively, these data clearly demonstrated that ORMDL3 regulates mitochondrial function and respiratory complex assembly. Dysfunctional respiratory complexes lead to higher mitochondrial reactive oxygen species (ROS) production. We also observed that after ORMDL3 knockdown, mitochondrial ROS production was significantly reduced in the human MDMs (Fig. 4*C*) validating the role of ORMDL3 in mitochondrial function.

ORMDL3 was shown before to regulate ER stress, which regulates mitochondrial morphology and function. To rule out the effect of altered ER stress on mitochondrial dynamics, we assessed mitochondrial morphology in the human MDMs after *ORMDL3* tampering and inhibiting/activating ER stress response pathways. ORMDL3 overexpressing human macrophages were treated with tunicamycin, an ER stress inducer which increases mitochondrial fusion at 6 h of treatment (32). Control cells treated with tunicamycin showed elongated mitochondria; however, *ORMDL3* overexpressing cells treated with tunicamycin showed more fissioned mitochondria suggesting that ORMDL3 mediated mitochondrial morphology alteration is independent of ER stress (Figs. 4*D* and S4*B*). We next checked the expression level of mitochondrial fission markers (Drp1) and fusion associated proteins (Mfn-1 and Mfn-2) and found that tunicamycin induced ER stress led to reduced Drp-1 level, with no change in mitofusin 1 and mitofusin 2 protein level (Fig. S4*C*). Interestingly, ORMDL3 depleted cells did not show this reduced Drp1 after tunicamycin treatment, albeit displayed higher mitochondrial fission.

Additionally, after inflammasome activation, ER stress inhibition using integrated stress response inhibitor (*trans*-ISRIB) or GSK2656157 in cells with *ORMDL3* overexpression displayed reduced IL-1β level, but was still significantly higher than the ISRIB- and GSK-treated control cells (Fig. 4*E*). GSK and ISRIB reduced ER stress response protein level including Phospho-eIF2 alpha, Chop, and Bip after tunicamycin treatment (Fig. S4, *D* and *E*) validating effective inhibition. These data collectively indicate that ORMDL3 mediated regulation of mitochondrial dynamics as well as IL-1β production has ER stress independent components.

### ORMDL3 binds to mitochondrial/MAM proteins

**Figure 5:**
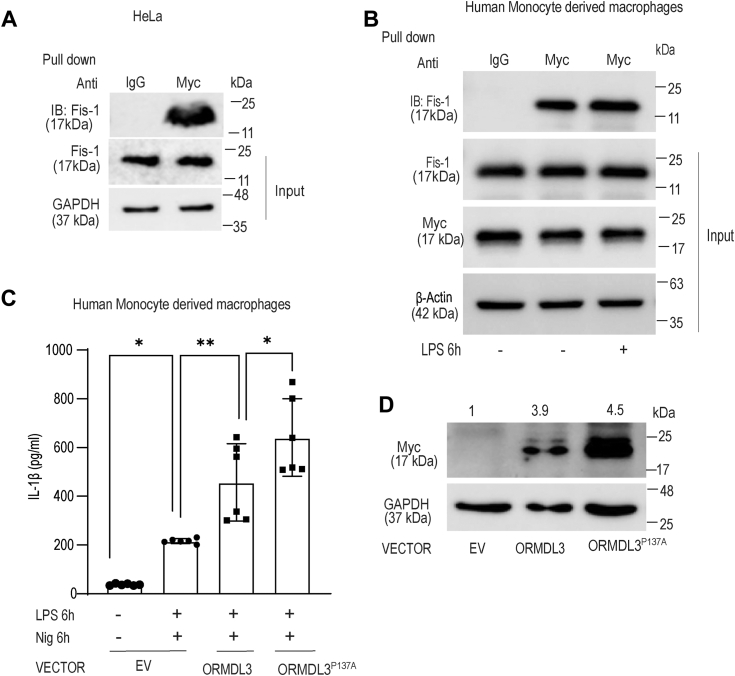
**ORMDL3 interacts with Fis1and interaction increases after LPS stimulation.***A*, HeLa cells were transfected with myc-tagged ORMDL3 expressing plasmid or empty vector for 24 h and total cell lysate was immunoprecipitated (pull-down) with anti-myc antibody and IgG control and accessed for interaction of ORMDL3 and endogenous Fis1, similar result obtained for additional repeat (n = 3). *B*, MDM cells were transfected with myc-tagged *ORMDL3* expressing plasmid and empty vector for 24 h, treated with LPS (250 ng/ml) for 6 h and total cell lysate immunoprecipitated with anti-myc antibody and IgG control and analyzed for interaction between ORMDL3 and Fis1 (n = 3). *C*, human MDMs were transfected with ORMDL3 proline 137 alanine mutant expressing plasmid (ORMDL3-P137A) or ORMDL3 full-length (ORMDL3) or empty vector (EV) for 24 h, treated with LPS and nigericin for 6 h and analyzed for IL-1β by ELISA (n = 6, similar result obtained for additional n = 6 donor). *D*, MDM were transfected with ORMDL3 proline 137 alanine mutant expressing plasmid or ORMDL3 full-length or empty vector for 24 h and analyzed for myc-ORMDL3 expression by Western blotting. Representative Western blot is shown; band intensity values normalized to GAPDH control are indicated above the band. Mean ± SEM; ∗*p* < 0.05; ∗∗*p* < 0.01; as determined by one-way ANOVA. IL, interleukin; LPS, lipopolysaccharide; MDM, monocyte-derived macrophage; ORMDL3, orosomucoid-like protein 3.

**Supporting Figure S5:**
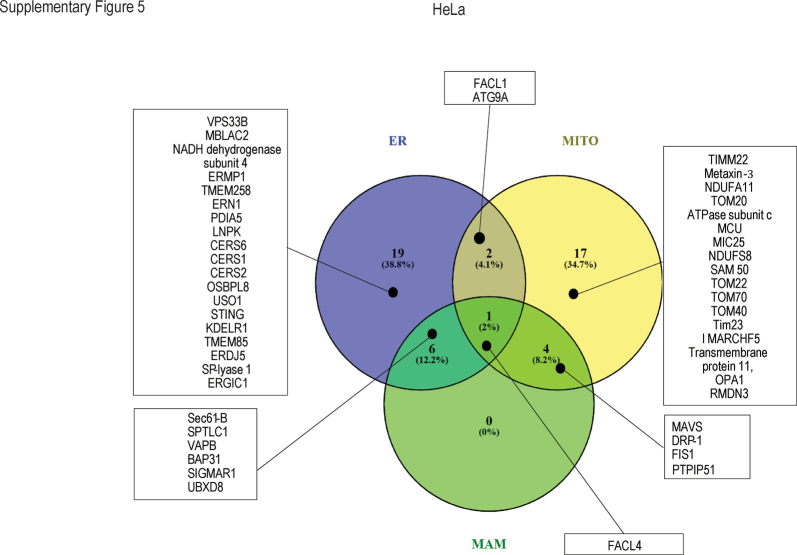
**ORMDL3 interactome analysis by mass spectrometry.** HeLa cells were transfected with Myc tagged ORMDL3 expressing plasmid or empty vector and total cell lysate was immunoprecipitated with anti-myc antibody and IgG control. IP samples were sent for mass spectrometry in triplicates. Venn diagram representing ORMDL3 interacting proteins present in ER, mitochondria and mitochondria associated membrane is shown.

The very fact that ORMDL3 is localized to the mitochondria and regulates MAM integrity and mitochondrial morphology, points to a possible physical interaction with some mitochondrial protein. We therefore performed mass spectrometry analysis after *ORMDL3* overexpression in the HeLa cells. Although ORMDL3 is a well-known ER-localized transmembrane protein, to our surprise multiple mitochondria and MAM proteins were obtained as putative binding partners (Fig. S5). Coimmunoprecipitations followed by immunoblotting was performed specifically for proteins from our mass spectrometry data which regulate mitochondrial dynamics (24, 33). *Myc* tagged *ORMDL3* in the HeLa cells showed interaction with one of the important player of mitochondrial fission- Fis1 (Fig. 5*A*). Interestingly, we observed interaction of ORMDL3 with Fis1 in the human MDM at both basal and LPS stimulated condition (Fig. 5*B*). Fis1 is recruited to the MAM during basal as well as several other conditions including hypoxia, inflammation, and promote mitochondrial fission (34). LPS treatment increases mitochondrial fragmentation and mitochondrial dysfunction where Fis1 is the key to the pathological damage to mitochondria (35). Our result suggests that Fis1 physically interacts with ORMDL3 and possibly this association helps in mitochondrial fission as well as enhanced ER-mitochondria tethering.

### ORMDL3 residue proline 137 is important for its stabilization and contributes to the inflammation

ORMDL3 has prolyl hydroxylase consensus sequence at C terminus, which is important for ubiquitination, extraction from the ER membrane, and degradation for the maintenance of ORMDL3 protein level. ORMDL3 residue proline 137 is important for ORMDL3 degradation and maintenance of ORMDL3 protein level (36). We substituted proline 137 with alanine (ORMDL3-P137A) and interestingly ORMDL3 P137A mutant when transfected in human MDMs showed higher IL-1β production compared to ORMDL3 full-length plasmid transfected cells, when stimulated with LPS and nigericin (Fig. 5*C*). We checked ORMDL3 protein level in MDM cells with overexpression of ORMDL3-P137A and observed that ORMDL3-P137A has indeed high protein expression compared to ORMDL3 full-length plasmid overexpression (Fig. 5*D*).

### ORMDL3 plays a crucial role in mounting inflammation in the animal dextran sodium sulfate–induced colitis model

Mice express three ORMDL family proteins with ORMDL3 having 96% similarity with its human counterpart (14). We chemically induced gut inflammation in mice by providing 2% dextran sodium sulfate DSS in the drinking water. This sulfated polysaccharide causes damage to the colonic epithelium and exhibits clinical features similar to observed in the ulcerative colitis, such as disruption of epithelial barrier integrity and colonic mucosal lesions (37). Additionally, DSS-induced colitis mice show enhanced IL-1β level produced by NLRP3 inflammasome (38).

**Figure 6:**
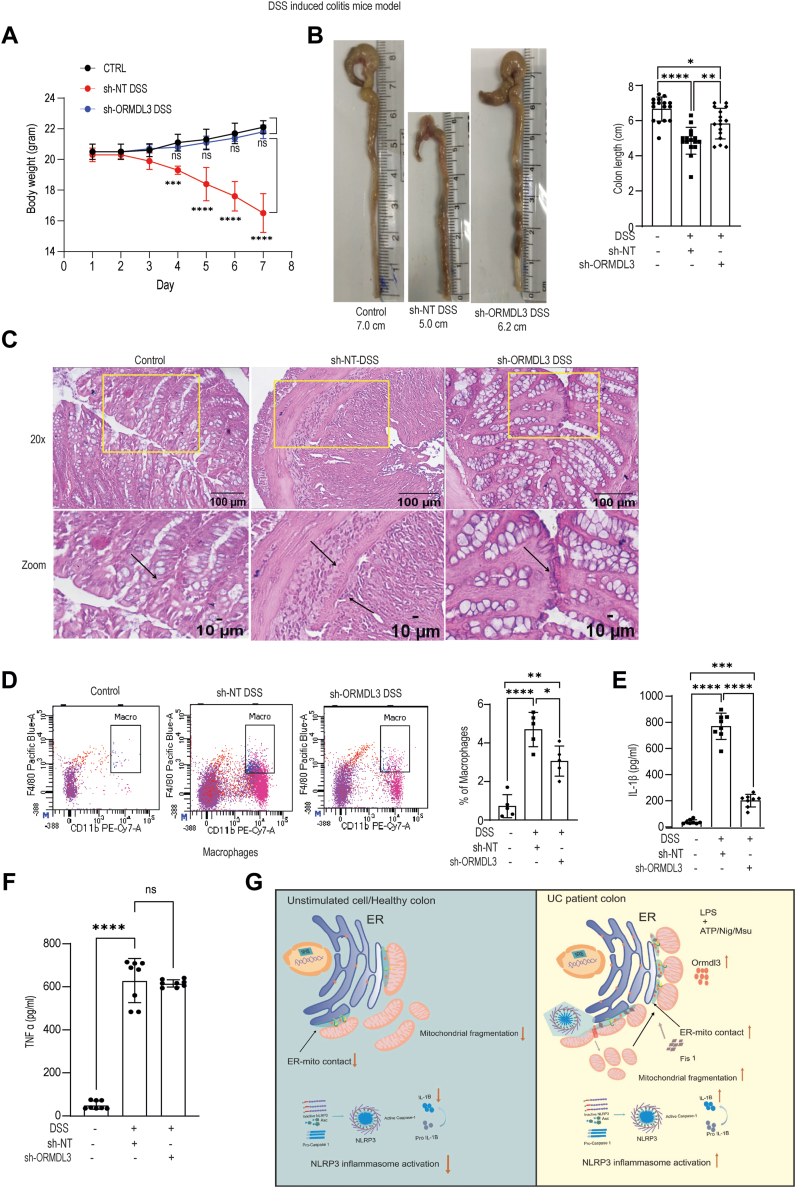
**ORMDL3 ablation reduces inflammation in the colon of DSS colitis mice characterized by decreased infiltration of inflammatory cells and proinflammatory cytokine.** C57BL/6 male mice (7-week-old) were fed 2% DSS in drinking water for 7 days to induce colitis. ORMDL3 shRNA or non-targeted shRNA expressing lentivirus was administered *via* i.p. route (5 × 10^6^ particles/mouse before DSS administration). *A*, comparative analysis of body weight change after DSS administration in each group (n = 5). *B*, comparison of colon length and summary graph showing colon length quantification (n = 16, pooled from three different experiment). *C*, analysis of histopathological changes in distal colon by haematoxylin and eosin (H&E) staining using Leica DMi-5000 microscope, 20X and zoom representative images from *yellow* highlighted are shown. Lamina propria cells (LPMC) were isolated from colon of mice in all groups and stained using antibody cocktail to stain immune cell population such as macrophages cells and other myeloid cells. *D*, representative histograms showing flow cytometry analysis of macrophages/homing in the lamina propria of different mice group and summary graph showing macrophage infiltration (n = 5). Colon tissue from all mice group(control, sh-NT-DSS, sh-ORMDL3-DSS) were lysed using lysis buffer and supernatants were analyzed for (*E*) IL-1β level (n = 8 mice), similar results were obtained for additional repeat (n = 8). *F*, TNF-α level (n = 8 mice). Similar results were obtained from n = 8 mice. *G*, pictorial model depicting effect of ORMDL3 expression in mitochondrial dynamics, ER-mitochondria contact in the ulcerative colitis patient. In UC patient, ORMDL3 expression increases causing elevated ER-mitochondria contact which in turn leads to mitochondrial fragmentation and NLRP3 inflammasome activation as well as IL-1β secretion. Mean ± SEM; ∗*p* < 0.05; ∗∗*p* < 0.01; ∗∗∗*p* < 0.001 ∗∗∗∗*p* < 0.0001; as determined by one-way ANOVA. DSS, dextran sodium sulfate; ER, endoplasmic reticulum; IL, interleukin; ORMDL3, orosomucoid-like protein 3; TNF, tumor necrosis factor; UC, ulcerative colitis.

**Supporting Figure S6:**
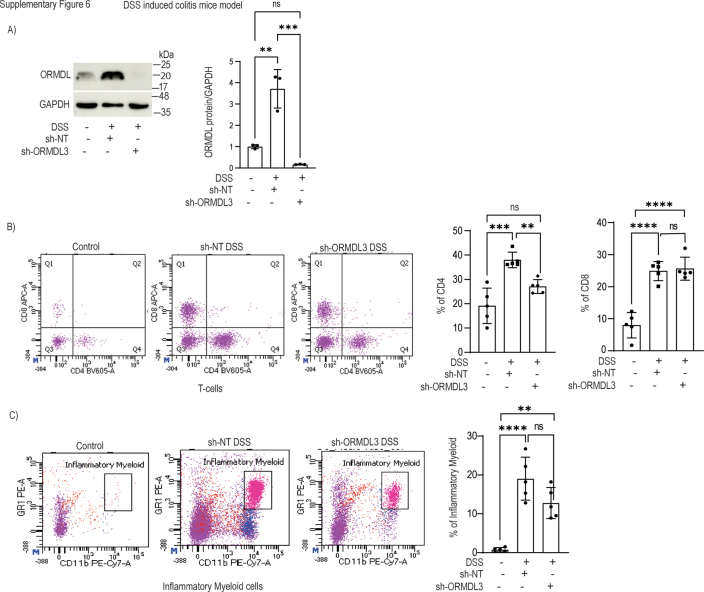
**ORMDL3 knockdown in mice intestine reduces infiltration of proinflammatory immune cells in the lamina propria.** C57BL/6 male mice (7 weeks old) were fed 2% DSS in drinking water for 7 days to induce colitis. ORMDL3 shRNA or non-targeted shRNA expressing lentivirus was administered *via* i.p. route (5 × 10^6^ particles/mouse before DSS administration). *A*, *ORMDL3* knockdown efficiency in mice colon by western blot and summary graph of densitometry is shown from 3 animals. GAPDH was used as loading control. Lamina propria cells isolated from mice colon and stained using antibody cocktail to stain immune cell population present in the lamina propria and analysed by flow cytometry (*B*) Histogram and summary bar graph showing T cell infiltration in the lamina propria from n = 5 animals (*C*) Histogram and summary bar graph showing Infiltration of proinflammatory myeloid cells in the lamina propria from n = 5 animals. Mean ± SEM; ∗∗*P* < 0.01; ∗∗∗*P* < 0.001; ∗∗∗∗*P* < 0.0001 as determined by one-way ANOVA. ns denotes non-significant.

To understand the role of ORMDL3 during DSS-induced colitis, we downregulated ORMDL3 expression in the animal using lentivirus containing *ORMDL3* shRNA injected *via* intraperitoneal route in mice. In order to access the role of ORMDL3 in the colitis formation, we organized the animal into three experimental groups namely, control, DSS treatment with nontargeted shRNA (DSS-sh-NT), and DSS treatment with *ORMDL3* shRNA (DSS-sh-ORMDL3). By a series of experiments, the severity of inflammatory response was next monitored. The efficiency of ORMDL protein knockdown is shown in Fig. S6*A*. First, we monitored the changes in body weight for seven consecutive days. The DSS-sh-NT group showed decrease in the body weight and by seventh day they were approximately 25% lesser compared to the control group. Whereas, the DSS-shORMDL3 group did not exhibit any significant reduction in body weight compared to the control group (Fig. 6*A*).

Next, we evaluated the reduction in colon length, a characteristic feature of DSS-induced colon inflammation. We observed that while DSS-sh-NT group showed significantly reduced colon length (Fig. 6*B*) than the control animals, the colon length of DSS-sh-ORMDL3 group showed a partial rescue. Following this, we carried out histological analysis to access the differences in the tissue architecture and inflammatory infiltrates. DSS-sh-NT group showed compromised colon epithelial architecture indicative of lesions (marked by arrows) whereas the DSS-sh-ORMDL3 had a significant rescue (Fig. 6*C*). Similar observations were also made by quantitating the population of immune cells in colonic lamina propria by flow cytometry analysis. Although DSS-sh-ORMDL3 exhibit presence of inflammatory cellular populations, clearly DSS-sh-NT showed highest number of macrophages (Fig. 6*D*). We also checked other inflammatory cells including T cells and inflammatory myeloid cells in the lamina propria in all the three mice groups. Both the immune cell populations were significantly high in DSS-sh-NT group when compared to the control; however, only CD4 positive T cells were significantly upregulated in the DSS-sh-ORMDL3 group (Fig. S6, *B* and *C*). We next assessed IL-1β and TNF-α level in the intestinal tissue sample of all the three groups. IL-1β level was significantly high in the DSS-sh-NT group than the control animals. IL-1β level in the DSS-sh-ORMDL3 group was higher than the control, but it was significantly reduced in comparison to the DSS-sh-NT group (Fig. 6*E*). TNF-α level also increased in the animal after DSS treatment. Importantly, TNF-α level in mice after ORMDL3 knockdown was similar to the DSS-sh-NT mice suggesting that ORMDL3 expression level did not affect TNF-α level during DSS-induced colitis (Fig. 6*F*). These data indicate that ORMDL3 knockdown significantly restores the DSS induced disease pathology.

Collectively, we thereby provide compelling evidence that ORMDL3 mediated altered ER–mitochondria contacts dictate UC pathogenesis by altering the immune response, specifically NLRP3 inflammasome activation and IL-1β secretion in the human macrophages and inflamed mucosa (Fig. 6*G*).

## Discussion

Unraveling the role of IBD GWAS-associated genes over the last decades have helped in understanding the functional relevance of genes contributing to major inflammatory pathways during IBD pathogenesis. Targeting the genes contributing to inflammatory pathway is the key to newer clinical treatment strategies in IBD (39). In this study, we have unveiled the role of IBD susceptibility gene, *ORMDL3* and identified its contribution to inflammatory axis. Interestingly we found that enhanced expression of *ORMDL3* in disease condition exacerbates inflammation by contributing to high IL-1β production (Fig. 6*G*).

Macrophages are the major contributor of inflammation and are the keys to initiation, maintenance, and resolution of inflammation in IBD. Activated macrophages function by antigen presentation, phagocytosis, and immunomodulation by producing various cytokines (40) which is a key pathology in IBD. Macrophage activation followed by exposure to bacterial LPS as well as proinflammatory cytokines is tightly regulated, as excessive activation is detrimental to the tissues (41). Macrophage prolonged activation in UC produces cytokines including IL-1β and TNF-α, which are keys to the inflammation loop (42). We have checked expression of *ORMDL3*, as well as IL-1β, and TNF-α level in the patients’ colonic biopsy sample. IL-1β level in the patients correlated with their ORMDL3 expression. Although TNF-α level were elevated in the patients, it did not show significant correlation with ORMDL3 expression. Since macrophages predominantly contribute to inflammation in the intestinal mucosa and submucosa of UC patients (43), we extrapolated our study in human MDMs.

Mitochondrial dysfunction is associated with UC pathogenesis. Further, exposure to bacterial ligand is a major contributor of IL-1β production in the patients (6). We have also found that ORMDL3 expression is upregulated in the LPS stimulated macrophages, validating previous study (44). Cells with *ORMDL3* upregulation has reduced OCR and elevated mito ROS indicating mitochondrial dysfunction. In response to various cellular physiological conditions, mitochondria makes close contact with other organelles like ER and provides platform for immune signaling and regulates mitochondrial morphology which is less explored in IBD (45). Our study found that in cells with ORMDL3 overexpression there is accumulation of this protein at ER-mitochondria contact which eventually leads to increased ER-mitochondria contacts. NLRP3 inflammasome activation and IL-1β release is a major pathology in the UC patients and also in the DSS-induced colitis model. Persistent inflammasome activation is termed causative for multiple chronic diseases including IBD (46). Our results showed that ORMDL3 also facilitates mitochondrial fission and NLRP3 inflammasome activation. Proteins present on the ER interact with mitochondrial proteins when ER is closely apposed to mitochondria and regulates mitochondrial dynamics (47). ER–mitochondria contact facilitates exchange of ions and metabolites such as lipids, glucose, and ROS between the organelles (48). Altered ER and mitochondria contact formation is reported during various pathological states. Further, mitochondrial morphology and localization determines ER–mitochondria contact formation as mitochondrial elongation leads to decreased ER–mitochondria contact.

ORMDL3 interaction with Fis1 might provide new insights into mitochondrial dynamics. Fis1 recruits multiple fission inducing proteins like Drp1 (49)) and TBC1D15 and helps in mitochondrial fission (50). Additionally, Fis1 physically interacts with ER resident protein BAP31 to bridge ER and mitochondria and form a platform for procaspase-8 recruitment during apoptosis induction (51). We believe that the interaction of ORMDL3 with Fis1 leads to ER–mitochondria tethering, enhanced MAM integrity which ultimately allows higher NLRP3 docking platform (52). It is of interest to note that a reduced Sigma1R in the total brain lysate correlates with MAM deficits in the DJ-1 KO animals (53). Additionally, MFN2 KO cells displayed higher ER-mitochondrial contacts and all the MAM proteins tested were enriched in the knockdown cells (54). Both these studies indicate that the MAM marker expression can be directly correlated with MAM integrity and our result showed upregulation of MAM marker FACL4 in cells with ORMDL3 overexpression; suggesting its role in MAM integrity and MAM mediated signaling.

UC patients with higher ORMDL3 in the colon also had enhanced IL-1β secretion. What could be the mechanism in the patients to upregulate ORMDL3? ORMDL3 polymorphisms in the patients might be the reason for enhanced expression as observed before for the asthma patients (55). Similar mechanism might be present in our cohort. The bacterial components present in the intestinal mucosa might lead to higher ORMDL3 transcription in the UC patients as observed after LPS stimulation in the macrophages in our study. Additionally, prolyl hydroxylation has been shown previously to degrade ORMDL3. *PHD1* mRNA expression which is downregulated in both the inflamed tissues derived from UC patients and in cultured intestinal epithelial cells treated with inflammatory cytokines (56), might also be the reason for higher ORMDL3 in the patients.

Our study thereby provides strong evidence that ORMDL3 can bind with MAM and mitochondrial compartments and this association plays a crucial role in mounting inflammatory response. This study for the first time provides mechanistic understanding for the genetic association of ORMDL3 gene with UC. ORMDL3 knockdown in DSS-induced colitis mice model showed significant reversal of the disease pathology. Moreover, proinflammatory cytokine IL-1β was attenuated after ORMDL3 knockdown, highlighting the possibility of using ORMDL3 as a new therapeutic target in UC. Understanding the functional relevance of ORMDL3 and specifically in regulating ER-Mitochondria contacts, NLRP3 inflammasome activation opens up new avenues for drug discovery.

## Experimental procedures

### Cell culture

Human primary MDMs and HeLa cells (American Type Culture Collection) were maintained in RPMI and Dulbecco's modified Eagle's medium supplemented with 10% fetal bovine serum, 2% penicillin/streptomycin/amphotericin. Cells were grown at 37 °C and 5% CO_2_. Subsequently, 1X trypsin–EDTA (prepared from 10X trypsin–EDTA) in PBS was used to detach adherent cells.

#### Reagents

The following reagents were used. Dulbecco's modified Eagle's medium (Gibco), RPMI (Gibco), GSK 2656157, PERK inhibitor (Cayman chemical), *trans*-ISRIB (Cayman chemical), Lipofectamine 3000 Transfection kit (Invitrogen), Percoll (Sigma-Aldrich), Clarity Western ECL substrate (Bio-rad), Protease Inhibitor Cocktail kit(MP Biomedicals), Pierce Protein A/G PLUS-Agarose (Thermo Fisher Scientific), XF Base Medium (Agilent Seahorse), Human IL-1β/IL-1F2 Duoset ELISA (R &D), Human IL-10 Duoset ELISA (R&D), Human TNF-alpha Duoset ELISA (R&D), Mouse IL-1β & TNF-alpha Duoset ELISA (R&D), RevertAid First Strand cDNA Synthesis Kit (Thermo Fisher scientific), Maxima SYBR Green/ROX qPCR Master mix (Thermo Fisher scientific), lipopolysaccharide (LPS, Sigma-Aldrich), MSU (InvivoGen), nigericin (Cayman Chemical), ATP (Cayman Chemical), Lipofectamine RNAiMAX reagent (Invitrogen), M-CSF Human (GenScript Biotech,), siRNA negative control (Dharmacon and Santa Cruz Biotechnology); siRNA ORMDL1, ORMDL2, ORMDL3 (Dharmacon and Santa Cruz Biotechnology).

### Antibodies and plasmids

Antibodies used for experiments are anti-beta Actin (Affinity, AF7018); anti MYC tag (ABclonal, AE070); anti calnexin (Cell Signaling Technology, C5C9); anti eIF2α (Cell Signaling Technology, D7D3); anti IP3R3 (Santa Cruz Biotechnology, 377518); anti Phospho-eIF2α (Ser51), (Cell Signaling Technology, D9G8); VDAC (Cell Signaling Technology, D73D12); anti-ORMDL3 (ABclonal, A14951); anti NLRP3 (Cell Signaling Technology D2P5E, D4D8T); anti MTCO2 (Abcam, 963298); anti FIS1 (ABclonal, A19666); anti DRP1,(Cell Signaling Technology D6C7); anti MFN2 (Cell Signaling Technology,D1E9); anti-ASCL4/FACL4 (Affinity, DF12141); anti-GAPDH,(Affinity, T0004#1825); immunoglobulin G (IgG) (H + L) Fluor594 (Affinity S0006) IgG (H + L) Fluor488 (Affinity, S0017).

### Plasmids used for experiments

ORMDL3-PCDNA myc-tagged (GenScript Biotech), ORMDL3 P137A, myc-tagged (GenScript Biotech).

### PBMC isolation and MDM preparation

Peripheral blood mononuclear cells were isolated from peripheral blood of healthy human donor by density gradient centrifugation using HiSep according to the manufacturer’s instruction. Blood and PBS mixture in 1:1 ratio overlayed on HiSep medium and centrifuged at 400*g* for 30 min at 25 °C. Mononuclear cells were isolated from layer formed above interphase of HiSep and pelleted. Red blood cell (RBC) contaminants present were lysed using RBC lysis buffer (0.15 M ammonium chloride, 0.01 M potassium bicarbonate, and 0.0001 M disodium EDTA). After incubation with RBC lysis buffer for 5 to 10 min’ cells were washed in PBS. Finally cells were resuspended in RPMI media and plated to adhere to monocytes. Monocytes adhered to culture plate were cultured in RPMI supplemented with 10 ng/ml macrophage colony-stimulating factor for 5 to 7 days to prepare macrophage (57).

### Human tissue sample collection

Tissue sample of UC patients were collected during colonoscopy by the gastroenterologists at Sanjay Gandhi Postgraduate Institute of Medical Sciences. Control samples were collected in the same way from colon biopsies taken from patients with irritable bowel syndrome. UC tissue samples were analyzed for ORMDL3 expression as well as cytokines analysis and correlated with the DAI of patient (19). DAI/DAI score assess the severity of disease in the patients. It ranges between 0 and 25 depending on parameters for evaluation. Patient is assigned a score based on objective (extraintestinal manifestations, number of stools per week, temperature, erythrocyte sedimentation rate, and hemoglobin) and subjective parameters (general well-being, blood in stool, and a sum of abdominal pain/cramps incidences in the previous week). Based on score, patients were categorized as remission (0–4), mild (5–10), moderate (11–17), and high (more than 18). The studies were approved by Central Drug Research Institute (CDRI) and SGPGI Institutional Ethics Committee review board.

### Transfections

siRNAs (100 nm) were electroporated using Amaxa Nucleofector (Amaxa) or transfected using lipofectamine RNAiMAX reagent according to the manufacturer’s instruction. Plasmid constructs and mutants were transfected using Lipofectamine 3000 Transfection kit according to the manufacturer’s instruction. Cells were cultured for 24 to 48 h at 37 °C and 5% CO_2_ after transfection prior to the treatment and lysis.

### Inflammasome activation

To check the role of ORMDL3 in NLRP3 inflammasome activation, ORMDL3 depleted as well as ORMDL3 overexpressing human blood-derived monocytes and MDMs were pretreated with LPS (200 ng/ml) for 6 h. To activate NLRP3 inflammasome, LPS treated cells were stimulated with 5 mM ATP additionally for 30 min whereas other stimulant including 150 μg/ml MSU and 10 μM nigericin were added along with LPS. NLRP3 inflammasome-activated cells were further analyzed by microscopy and Western blot to assess NLRP3 localization to mitochondria and ELISA to assess IL-1β secretion.

### Western blotting

Cells were lysed in radioimmunoprecipitation assay buffer or NP40 lysis buffer supplemented with protease inhibitor cocktail, lysate were centrifuged at 12,000*g* for 15 min at 4 °C. Supernatants after centrifugation were boiled with 1X Laemmli buffer and same concentrations of the proteins were resolved using SDS-PAGE. Proteins were transferred on polyvinylidene fluoride membrane and incubated with primary antibody overnight at 4 °C. Primary antibodies against ORMDL3 (1:1000), Myc (1:1000), Fis1(1:1000), DRP1 (1:1000), FACL4 (1:1000), VDAC (1:1000), calnexin (1:1000), phospo-eIF2alpha (1:1000), Eif2-alpha (1:1000), MFN2(1:1000), and NLRP3 (1:1000) were used. Anti-GAPDH (1:1000), and anti-beta actin (1:1000) antibodies were used as loading control. Horseradish peroxidase–conjugated secondary antibodies against rabbit (1:10,000) and mouse (1:10,000) were used. Band intensity was quantified by ImageJ (https://imagej.net/) software.

### RNA isolation and mRNA expression analysis

Total RNA was extracted from cells and tissues (human and mice) using TRIzol reagent according to the manufacturer’s instruction. RNA was reverse transcribed using RevertAid First Strand cDNA Synthesis kit according to the manufacturer’s instruction. Quantitative PCR was performed using SYBR Green master mix to assess gene expression using gene specific primers. GAPDH/and β-2-microglobulin were used as control.

### Caspase 1 activation assay

Caspase-1 activation in the human MDM was checked by FAM-FLICA caspase assay kit (ImmunoChemistry Technologies) after ORMDL3 knockdown in the human MDMs according to the manufacturer’s instructions. Cells were stimulated with LPS and nigericin to activate NLRP3 inflammasome. Caspase 1 activation was assessed by flow cytometry (FACSAria II cell sorter, BD Biosciences).

### Subcellular fractionation

Mitochondria-associated membrane, mitochondria, and ER were isolated from the HeLa cells as described before (58). Briefly, cells grown in 90 mm Petri dish to confluency were detached using 1X trypsin. Cells were pelleted at 600g for 5 min at room temperature and washed in PBS twice. Cells were resuspended in isolation buffer (sucrose, mannitol, and EGTA) and homogenized. Unbroken cells and nuclei were removed by centrifugation at 600*g* for 10 min at room temperature twice. Crude mitochondria were isolated by centrifugation at 10,000*g* for 10 min at 4 °C. Cytosol was centrifuged at 100,000*g* for 1 h at 4 °C after removal of crude mitochondria to isolate ER. Crude mitochondria were resuspended in mitochondria resuspension buffer and overlayed on 30% percoll medium; mitochondria and mitochondria-associated membrane were segregated by ultracentrifugation at 95,000*g* for 30 min at 4 °C. Further mitochondria pellets at base were washed and pelleted at 6300*g* for 10 min at 4 °C and mitochondria-associated membrane present as diffused band at top were pelleted at 10,000*g* for 1 h at 4 °C. Expression of ORMDL3 was analyzed in total cell lysate, ER, mitochondria, and mitochondria-associated membrane and purity of the various fractions were checked using anti-VDAC (mito), anti-calnexin (ER and MAM), and anti-FACL4 (MAM) antibodies.

### Transmission electron microscopy

HeLa cells were transfected with empty vector or ORMDL3 overexpression vector and cells were visualized using TEM to check the effect of ORMDL3 on ER-mitochondria contact. Briefly, HeLa cells were fixed with 2.5% glutaraldehyde for 24 h. Afterward, cells were rinsed with phosphate buffer, post fixed in osmium tetroxide, and encapsulated in agarose. The dehydration process was completed in a gradient ethanol series before the epoxy resin embedding process. For 24 h, the resin was polymerized at 60 °C. A LEICA EM UC7 ultramicrotome was used to cut ultrathin sections (50–70 nm), which were then collected on copper grids and stained with uranyl acetate and lead citrate. The grids were then studied at a 100 kV accelerating voltage using a JEOL JEM-1400 TEM equipped with a Gatan bottom-mounted Orius CCD camera.

### Proximity ligation assay

PLA was performed to analyze vicinity between ER and mitochondria, in HeLa cells transfected with empty vector and ORMDL3 overexpression vector using IP3R to label ER and VDAC to label mitochondria. PLA was done using Duolink *In situ* Red Starter kit mouse/Rabbit (Sigma-Aldrich DUO92007) according to the manufacturer’s instruction. Briefly, HeLa cells grown on coverslips were transfected with ORMDL3 overexpressing or empty vector. Subsequently, 24 to 48 h post transfection, cells were fixed, permeabilized, and incubated with primary antibody VDAC (1:100) (rabbit-D73D12) and IP3R (1:50) (mouse-sc377518) diluted in PBS + 0.1% Triton X-100 + 4% bovine serum albumin, overnight at 4 °C. Anti-rabbit and anti-mouse secondary IgG antibody conjugated with PLUS and MINUS oligonucleotides were added to the cells and incubated for 1 h at 37 °C. After ligation and amplification (carried as per manufacturer’s instruction), coverslips were mounted using Duolink mounting medium, and proximity of protein (<40 nm) were captured as red fluorescent dots using OLYMPUS BX61-FV1200-MPE. Coverslips without incubation with the primary antibodies were processed in a similar way as the control. Experiment was performed in duplicate and more than 600 cells were imaged. Average PLA dots were quantified using ImageJ software as dots per nucleus.

### Mice and *in vivo* studies

C57BL/6J WT male mice were obtained from CSIR-CDRI (Lucknow, India) animal facility and maintained under pathogen-free conditions on a 12-h light/12-h dark cycle. Animals were given chow and water *ad libitum,* 6 to 8 weeks old mice, weighing 18 to 25 g were used for experiment. Mice were segregated into three groups. To understand the role of ORMDL3 on colitis development, lentivirus (5 × 10^6^ particles/mouse) expressing ORMDL3 shRNA was administered *via* intraperitoneal route to reduce ORMDL3 expression 7 days before DSS administration in the experimental group. Nontargeted shRNA was administered in another group. Colitis was induced in both the groups by providing 2% DSS (36–50 kDa; MP Biomedicals) in the drinking water; however, control group was left untreated. In all the animal groups change in body weight and stool consistency were monitored daily after the induction of colitis. Mice were sacrificed after 7 days, and colonic tissues were collected for analysis. Colonic inflammation was assessed by measuring the infiltration of immune cells in the lamina propria of colon, histological damage, and cytokine analysis. The animal studies were approved by CDRI Institutional Animal Ethics Committee review board.

### Histopathological changes

To analyze histopathologic changes, mice colon was embedded in paraffin wax and resected into small sections. Paraffin embedded sections were then stained using haematoxylin and eosin and imaged under light microscope (Leica DMi-5000 microscope).

### Colonocyte isolation and flow-cytometry

Colonic lamina propria cells were isolated from mice large intestine according to the previously established protocol (59). Lamina propria cells were analyzed by flow cytometry to observe infiltration of the innate immune cells. Antibody cocktail of CD45-perCPcy5.5, MHC-II FITC, Gr-1-PE, CD11c- Texas red, CD11b-Pe Cy7, CD4-SB600, CD8-APC, and F4/80-PE (BD Bioscience) in magnetic-activated cell sorting buffer (Miltenyi Biotec) were used to stain macrophages, T cells, and other proinflammatory cells.

### Cytokine analysis

TNF-α, IL-1β, and IL-10 cytokine levels were assayed using ELISA kit from R&D system as per the manufacturer’s instructions from the human UC as well as non-UC colon biopsy samples, mice colon tissues, and cell supernatants. Tissue samples of human and mice were lysed in RIPA lysis buffer, and supernatants were collected. Human MDMs were stimulated with LPS (200 ng/ml for 24 h) or LPS along with ATP, MSU, and nigericin (200 ng/ml LPS, 150 μg/ml MSU, 10 μM nigericin, and 5 mM ATP for 6 h) at 24-48 h post transfection of *siRNA* (scrambled or siORMDL1/2/3). Supernatants after lysis of tissue samples as well as LPS and NLRP3 inflammasome stimulation were subjected to ELISA for TNF-α, IL-10, and IL-1β.

### Immunoprecipitation

HeLa cells/MDM were transfected with myc-ORMDL3 overexpression plasmid in 90 mm Petri dish. Subsequently, 48 to 72 h post transfection cells were lysed in NP40 lysis buffer. Supernatants (containing 1–2 mg protein) after centrifugation at 12,000*g* for 15 min at 4 °C incubated with anti-myc antibody overnight at 4 °C. Protein A Agarose bead added for 4 to 6 h (Invitrogen) to pull-down the protein complex. Beads were washed and protein eluted in 2X Laemmli buffer (without bromophenol blue). Immunoprecipitation samples were subjected to Western blotting and mass spectrometry to analyze ORMDL3 interacting proteins.

### Mass spectrometry

Protein samples were digested and reduced with 5 mM tris(2-carboxyethyl)phosphine and further alkylated with 50 mM iodoacetamide and then digested with trypsin (1:50, trypsin/lysate ratio) for 16 h at 37 °C. Digests were cleaned using a C18 silica cartridge to remove the salt and dried using a speed vac. The dried pellet was resuspended in buffer A (2% acetonitrile and 0.1% formic acid). Experiments were performed on an Easy-nlc-1000 system coupled with an Orbitrap Exploris mass spectrometer. A total of 1 μg of peptide sample was loaded on C18 column 15 cm, 3.0 μm Acclaim PepMap (Thermo Fisher Scientific) and separated with a 0 to 40% gradient of buffer B (80% acetonitrile and 0.1% formic acid) at a flow rate of 300 nl/min and injected for mass spectrometry analysis. Liquid chromatography gradients were run for 60 min. MS1 spectra were acquired in the Orbitrap (Max IT = 25 ms, AGQ target = 300%; RF Lens = 70%; R = 60K, mass range = 375 − 1500; Profile data). Dynamic exclusion was employed for 30 s excluding all charge states for a given precursor. MS2 spectra were collected for top 12 peptides. MS2 (Max IT = 22 ms, R = 15K, AGC target 200%). All samples were processed, and RAW files generated were analyzed with Proteome Discoverer (v2.5, https://knowledge1.thermofisher.com/Software_and_Downloads/Chromatography_and_Mass_Spectrometry_Software/Proteome_Discoverer/Proteome_Discoverer_Operator_Manuals/Proteome_Discoverer_2.5_overview) against the Uniprot Human database.

### Mito ROS detection

Mitochondrial ROS generation (superoxide levels) was detected using MitoSOX fluorescent marker (Life Technologies). Human MDMs were transfected with *siRNA* (*scrambled* or *siORMDL3)* for 24 h and stimulated with LPS (200 ng/ml) for 6 h post transfection. After LPS treatment, cells were incubated with 100 nM MitoSOX at 37 °C for 30 min, cells were washed and analyzed by flow cytometry (FACSAria II cell sorter, BD Biosciences).

#### Mitochondrial respiration

Mitochondrial OCR was measured after ORMDL3 knockdown and overexpression to analyze the effect of ORMDL3 expression on mitochondrial respiration using Agilent Seahorse XF24 Cell Mito Stress kit. Cells after ORMDL3 knockdown and overexpression as well as control cells were seeded in Seahorse XF24 cell culture microplates overnight (30,000 cells/well). Sensor cartridge was hydrated in Seahorse calibrant at 37 °C in a non-CO_2_ incubator overnight. Assay medium (supplemented with 10 mM glucose, 2 mM glutamine, and 1 mM sodium pyruvate) was added to the overnight grown cells and incubated for 45 min in a non-CO_2_ incubator. OCR was measured using Seahorse XF24 extracellular flux analyzer (Seahorse Biosciences, Agilent). Mito-stressors 1.5 μM oligomycin, 2 μM carbonyl cyanide-p-trifluoromethoxyphenylhydrazone (FCCP), and 0.5 μM Rotenone/Antimycin A diluted in assay medium were used. After the run, cells were collected in 50 μl 1N NaOH, and protein concentration was estimated by Bradford method.

#### Microscopy

To assess mitochondrial dynamics, human MDMs/HeLa cells were transfected with myc-tagged ORMDL3 plasmid or empty vector. Twenty-four hours post transfection, cells were fixed and stained. Mitochondria and ORMDL3 were stained with anti- MTCO2 antibody (Abcam), and anti- Myc antibody (proteintech) and nuclei were stained with 4′,6-diamidino-2-phenylindole (DAPI) (Acros Organics). Imaging was conducted with OLYMPUS BX61-FV1200-MPE microscope. ImageJ and Fiji (https://imagej.net/software/fiji/downloads) software were used to analyze mitochondrial length.

#### Quantification and statistical analysis

Statistical analysis was performed using GraphPad Prism (https://www.graphpad.com/features). *p* value was assessed by 2-tailed student’s test or 1-way/2-way ANOVA, *p* < 0.005 was considered significant. The results were expressed as mean ± SEM. The sample size and independent repeats are indicated in figure legends. Quantification of Western blot and microscopy were performed using image J and fiji. Quantification of mitochondrial length was done according to previous study (60, 61).

### Statement on human studies

Human studies in the manuscript abide by “Declaration of Helsinki” principles.

## Data availability

All data are contained within the manuscript. LCMS data and oligo sequence information are available upon request to the corresponding author.

## Supporting information

This article contains supporting information.

## Conflict of interest

The authors declare that they have no conflicts of interest with the contents of this article.
